# Effect of Storage Temperature on Selected Strength Parameters of Dual-Cured Composite Cements

**DOI:** 10.3390/jfb14100487

**Published:** 2023-09-22

**Authors:** Joanna Giełzak, Agata Szczesio-Wlodarczyk, Kinga Bociong

**Affiliations:** 1 Department of Prosthodontics, Medical University of Lodz, 92-213 Łódź, Poland; 2 University Laboratory of Materials Research, Medical University of Lodz, 92-213 Łódź, Poland; 3 Department of General Dentistry, Medical University of Lodz, 92-213 Łódź, Poland

**Keywords:** dual-cured composite cement, diametral tensile strength, three-point bending strength, modulus of elasticity, Vickers hardness, strength properties, composite cements

## Abstract

Direct restorations are currently the most popular restorations used in dental prosthodontics. Due to the increased requirements for materials used in the fabrication of fixed restorations, there is a need for evaluation of strength parameters of these materials, including dental cements. The present study investigated the change in selected strength parameters of four dual-cured composite cements as a function of storage temperature. The following were investigated: three-point flexural strength (FS), flexural modulus in bending (FM), diametral tensile strength (DTS) and Vickers hardness (HV). Four dual-cured composite cements were tested, i.e., Multilink Automix (Ivoclar Vivadent), seT PP (SDI), MaxCem (Kerr), and Bifix Hybrid Abutment (VOCO). Each of the tested cements was stored for 7 days at one of the selected temperatures: 8 °C, 15 °C, 25 °C, or 35 °C, before the samples were made. Strength properties (DTS, FS) are not strongly dependent on the storage temperature in the range of 8–35 °C. Some statistical differences were observed between the hardness of MaxCem and Multilink Automix storage in various temperatures. FS and FM were lowest for Bifix Hybrid Abutment, MaxCem and Multilink Automix storage at 25 °C, and highest for Bifix Hybrid Abutment, MaxCem, and seT PP stored in 35 °C. The cement with the highest filler content (70% by weight) showed the highest FS and HV.

## 1. Introduction

The multiplicity of materials for cementing indirect restorations guarantees their wide application in prosthetics. Any prosthetic work, regardless of the material from which it is made, can be effectively connected with a pillar in the cementation process. Additionally, the requirements for dental materials are steadily increasing, driven by expectations set by clinicians themselves. Since dental composites are one of the most well-studied groups of dental materials, it is also apparent that there is a great deal of variation in these tests, depending on the choice of material and/or its selected properties, as well as the way samples are prepared for testing [1,2]. In the case of most cements currently used for indirect restorations in dental prosthetics, the storage temperature recommended by manufacturers is room temperature. The authors of the study wanted to explicitly test the clinical aspect of storing prosthetic cements, and the consequences of storing them at temperatures outside the range provided by the product data sheet. Analyzing changes in strength parameters at different storage temperatures could give a clue to the prognosis for the use of cemented indirect restorations in patients [3].

Tests of composite cements are most often concerned with temperature, in the broadly defined range of room temperature, and the mechanical and physical properties. These include hardness, microhardness, compressive strength, three-point bending strength and modulus of elasticity as well as polymerization shrinkage and conversion rate.

Studies on the effect of heating composite cements on changes in their strength properties are of great interest to researchers. However, they involve different storage temperatures, without defining clear criteria for their selection [1,2,3]. Most studies confirm that heating composite cements results in significant improvements in their physical and strength properties [4,5,6,7,8,9,10,11]. There are also reports on the effect of cooling on the strength properties of materials; however, such studies are not as numerous as those considering the effect of heating on the properties of these materials [12].

In the literature, there are also studies comparing the strength properties of composite cements with composite materials used in restorative dentistry, at different storage temperatures. These studies show that heated composite material has a higher compressive strength, flexural strength, hardness, and modulus of elasticity compared to dual resin cements [13,14]. There are also complex and dynamic strength property testing systems that can be applied to dental cements. Combining sub-terahertz in situ spectroscopy and neutron methods with differential scanning calorimetry (DSC) allows for structural, energetic, and dynamic unraveling of the complex bond-reaction processes of GIC dental composites at the atomic level [15].

A significant problem is the ambiguous determination of temperature criteria in research. This may be due to the sheer difficulty in defining optimal storage conditions. Manufacturers of dental materials often present general storage conditions for a given material in the product data sheet, frequently allowing a large degree of latitude in interpreting the storage temperature range. Additionally, there is a considerable ambiguity in the very definition of room temperature, and, therefore, the recommended storage conditions for composite cements, as well as other dental materials are often undefined.

Room temperature, as defined by the Merriam-Webster dictionary, refers to the range from 15 °C to 25 °C (59–77 °F, 288–298 K) and describes this temperature as indicated for human habitation. Scientific organizations, such as the U.S. Environmental Protection Agency (EPA) and the International Union for Pure and Applied Chemistry (IUPAC) define room temperature as normal, i.e., 25 °C (77 °F, 298 K) [16]. There are also known reports by researchers highlighting the impact of improper storage of dental materials due to unconscious omission or error. It is believed that the human factor is the main factor leading to irregularities in storage of materials [17,18,19].

The relatively wide range of room temperatures and different manufacturers’ recommendations concerning storage conditions may be the main reason for the lack of comprehensive studies on the strength properties of composite cements. The wide variety of available materials used for cementing fixed prosthetic restorations suggests the need for a thorough study and systematization of the influence of physical factors on their strength parameters. Four commercially available dual-cured dental composite cements, used for setting indirect restorations, were selected for the study. The choice was driven by the need to conduct a study on a universal group of cements that are most commonly used by clinicians today. An additional advantage of dual composite cements is the simple application procedure, which translates into reproducibility in use. The dual catalytic system is applied where access to a large amount of light is difficult, and thus the polymerization process must be supported by the presence of an autopolymerization catalyst. Most current fixed restorations, due to their thickness, do not allow enough light to penetrate, which is why cements with dual bonding characteristics are popular among clinicians.

In order to perform a comprehensive analysis of the effect of storage temperature on the strength properties of composite cements, four dual-cured composite cements were selected and stored at four temperatures from 8 °C to 35 °C. The null hypothesis tested was that temperature has no effect on selected strength characteristics of dual-cured composite cements.

## 2. Materials and Methods

The subjects of the present study were four selected dual-cured composite cements: Multilink Automix (Ivoclare Vivadent), seT PP (SDI), MaxCem (Kerr), and Bifix Hybrid Abutment (Voco). The chemical compositions of the investigated cements are presented in Table 1.

Before the samples were made, the cements were stored for 7 days at one of the selected conditioning temperatures: 8 °C, 15 °C, 25 °C and 35 °C. An incubator (Adler—Camry, Warsaw, Poland) was used to store samples at 15 and 25 °C. Temperature continuity was confirmed by measurements with a thermometer inside the laboratory incubator. The samples stored at 35 °C were placed in an incubator DZ-2BCII Vacuum Drying Oven (ChemLand, Stargard Szczeciński, Poland).

Diametral tensile strength (DTS), Vickers hardness (HV), three-point flexural strength (FS), and flexural modulus (FM) were tested for each study group.

Diametral tensile strength (DTS) was tested by preparing 11 specimens, cylindrical in shape and measuring 6 mm in diameter and 3 mm in height, for each temperature group and each material. The compressing force of the specimens along a roller sidewall was measured until failure. The speed of displacement of the transverse beam was 2 mm/min. The test was carried out using a Zwick Roel Z020 testing machine (Zwick-Roel, Ulm, Germany).

Vickers hardness (HV) was tested on four randomly selected DTS specimens. Eleven measurements were made for each temperature group and for each material. The test was carried out on a Zwick ZH*µ* device (Zwick-Roell, Ulm, Germany). The static load was 10 N, and the penetration time was 10 s.

Three-point bending (FS) strength and flexural modulus by bending (FM) were tested by preparing seven specimens of 25 mm × 2 mm × 2 mm per material, for each temperature group. This test was carried out also on a Zwick Roel Z020 testing machine. The crosshead speed was 1 mm/min.

The exposure time of the specimens was carried out each time according to the product data sheet, for each of the cements tested. The cements were exposed to THE CURE TC-01 polymerization lamp from SPRING (Norristown, PA, USA), with a real power of 1200 mW/cm^2^, emitting light in the range from 450 to 490 nm.

Specimens used for DTS and HV testing were exposed on both sides according to the times specified in the product data sheets, while those used for FS and FM were exposed according to the following scheme: the entire specimen, then at three locations, and additionally on the other side at three locations, also following the times specified by the manufacturer in the product data sheets.

Each time after the samples were made, the samples were stored for 24 h in distilled water, in the incubator, at 37 °C until testing.

Statistical analysis for the samples was performed using Statistica version 13 software (StatSoft, Krakow, Poland). The obtained data were analyzed using the Shapiro–Wilk test to assess the normality of distributions, followed by the non-parametric Kruskal–Wallis test with multiple comparisons of mean ranks or one-way ANOVA (parametric test) with post hoc test (Tukey). The level of significance adopted was 0.05. The results were reported as mean values with standard deviation (SD) or medians with interquartile range (IQR) based on the distribution and homogeneity of variance.

## 3. Results

Table 2 presents the mean values with standard deviation (SD) or medians with interquartile range (IQR), based on a statistical analysis of three-point flexural strength (FS), flexural modulus (FM), diametral tensile strength (DTS), and Vickers hardness (HV) of Multilink Automix at 8, 15, 25 and 35 °C.

The flexural strength of Multilink was in the range of 91.8 MPa (25 °C)–105.2 MPa (15 °C). The Multilink DTS was between 44.1 (8 °C) and 46.2 MPa (25; 35 °C), and HV ranged from 38 (35 °C) to 46 (8 °C). No statistical difference was observed in FS or DTS results (Table 2). There were statistical differences in the FM results between 8 °C and 15 °C (*p* = 0.000473) (a); 8 °C vs. 35 °C (*p* = 0.002581) (b); 15 °C vs. 25 °C (*p* = 0.000175) (c); and 25 °C vs. 35 °C (*p* = 0.000224) (d). Statistically significant differences found in the HV values for Multilink were the following: 8 °C vs. 15 °C (*p* = 0.003546), and 8 °C vs. 35 °C (*p* = 0.002223).

Table 3 presents the mean values with standard deviation (SD) or medians with interquartile range (IQR) based on a statistical analysis of three-point flexural strength (FS), flexural modulus (FM), diametral tensile strength (DTS), and Vickers hardness (HV) of seT PP at 8, 15, 25 and 35 °C.

For seT PP cement, the highest FS value was observed at 35 °C (63.4 MPa), and the lowest at 8 °C (46.5 MPa). The DTS of seT PP was in the range 40.9 MPa (35 °C)–45.5 MPa (25 °C). HV ranged from 19 (8 °C) to 24 (25 °C). The highest FM value was observed at 35 °C (3070 MPa), and the lowest FM at 8 °C (2300 MPa). No statistical difference was observed for FS, FM and DTS (Table 3). Significant statistical differences were found in the HV results for seT PP between 8 °C and 25 °C (*p* = 0.25038) (a).

Table 4 presents the mean values with standard deviation (SD) or medians with interquartile range (IQR) based on a statistical analysis of three-point flexural strength (FS), flexural modulus (FM), diametral tensile strength (DTS), and Vickers hardness (HV) of MaxCem at 8, 15, 25 and 35 °C.

The flexural strength of MaxCem was in the range of 73.1 MPa (8 °C)–85.8 MPa (35 °C). DTS values ranged from 49 MPa (35 °C) to 56.3 MPa (15 °C). The highest FM value was observed at 35 °C (6110 MPa), and the lowest at 25 °C (5032 MPa). The lowest HV was 30 (25 °C), whereas the highest was 39 (8 °C) (Table 4). Significant statistical differences were found for DTS results between 15 °C vs. 25 °C (*p* = 0.037021) (a) and 15 °C vs. 35 °C (*p* = 0.018694) (b). There were also statistical differences in HV results: 8 °C vs. 15 °C (*p* = 0.000350) (a); 15 °C vs. 25 °C (*p* = 0.036401) (b); 15 °C vs. 35 °C (*p* = 0.025057) (c); 8 °C vs. 25 °C (*p* = 0.000163) (d); and 25 °C vs. 35 °C (*p* = 0.00166) (e).

Table 5 presents the mean values with standard deviation (SD) or medians with interquartile range (IQR) based on a statistical analysis of three-point flexural strength (FS), flexural modulus (FM), diametral tensile strength (DTS), and Vickers hardness (HV) of Bifix Hybrid Abutment at 8, 15, 25 and 35 °C.

The flexural strength of Bifix Hybrid Abutment was from 94 MPa (25 °C) to 120 MPa (35 °C). The DTS of Bifix Hybrid Abutment was in a range of 43.9 MPa (8 °C)–54.8 MPa (25 °C). HV values ranged from 54 (8 °C) to 56 (15; 25; 35 °C). The highest FM value was observed at 15 °C (8760 MPa), whereas the lowest FM value was 6050 MPa (25 °C). Significant statistical differences were found in the FS results for Bifix Hybrid Abutment cement between 15 °C and 25 °C (*p* = 0.022181) (a), and between 25 °C and 35 °C (*p* = 0.001800) (b). There were also statistical differences in the FM results: 15 °C vs. 25 °C (*p* = 0.001050) (a), and 25 °C vs. 35 °C (*p* = 0.021836) (b).

## 4. Discussion

The study aimed to analyze how the storage temperature of dual-cured composite cements can affect their strength parameters. The constancy of properties in broadly understood room temperature conditions seems very important for the durability and success of prosthetic treatment. Additionally, the influence of cooling (8 °C) and heating (35 °C) of selected dual-cured composite cements was assessed. Considering the obtained results, the assumed null hypothesis cannot be unequivocally rejected. Statistically significant differences were not observed between the selected temperatures for most materials and the tests performed. The study observed that the DTS values for all of the tested cements were in the range of 40–56 MPa, which is consistent with the study of Franc et al., who also examined the effect of temperature on the tensile strength of cements, i.e., RelyX ARC and Variolink II [20]. However, in the literature, there are no studies that have tested strength parameters over a larger temperature range on dual composite cements alone. Most researchers have dealt with the change in strength parameters of resin cements or composite materials used in restorative dentistry as applied to dental prosthetics [21,22,23,24]. Sokolowski et al. conducted DTS studies of selected resin-based cements (self-adhesive and self-etching). Seventeen different cements were selected for the study. It was found that the average DTS values of the tested cements were also similar, in the range of 26–59 MPa [25]. Comparable values for DTS were obtained by Kim et al., who conducted tests on Relay X U200, G-Cem and LinkAce cements [26]. DTS in the present study reached the highest values for Bifix Hybrid Abutment cement at the storage temperature of 25 °C, for MaxCem cement at 15 °C, Multilink Automix at 35 °C, and seT PP at 15 °C.

DTS can reach similar values for different materials, and this is due to a number of factors that can act synergistically. These factors are the following: the size of the filler and its content in the material, the structure of the polymer matrix, and also the interaction between the matrix and the filler itself [25]. The results for the DTS values can only give some clues for conclusions. Therefore, different strength features of materials should be checked. DTS values themselves were highest for Bifix cement. The above observations apply to all temperature groups. In this study, it was found that the highest DTS stability was recorded for Multilink cement (results for different groups were most similar to each other). However, in the seT PP and Bifix Hybrid Abutment materials, no significant statistical differences were observed between DTS values at different temperatures, which suggests that different storage temperatures have no effect on the DTS of dental cement.

Another important parameter examined in the present study was the three-point flexural strength (FS). This property appears to be of great clinical importance, since most of the forces in the oral cavity are analogous to bending. The three-point flexural strength, which is the maximum stress observed when the specimen is bent before failure, reached the lowest values for each of the selected cements at 25 °C, except for seT PP cement, which reached the lowest value for FS at 8 °C. The highest values for FS were achieved at 35 °C for Bifix Hybrid Abutment (120 MPa), MaxCem (85.8 MPa) and seT PP (63.4 MPa). The study by Katayama et al. was conducted on five commercial resin composites selected to present varying degrees of filler content. The samples in the study were stored for 7 days at 37 °C, and then FS was measured. These results were compared with the values obtained for the control group, where the samples were stored for 7 days at room temperature. No correlation was found between the inorganic filler content and the storage temperatures of the tested cements. It was concluded that changes in FS may be multifactorial. The change in FS can be influenced by parameters of the resin composite for provisional restorations, such as the types of filler along with its content, the composition of the monomer in the resin matrix, and even the addition of plasticizer [27].

The highest value for three-point bending (FS) for Multilink Automix cement was obtained at 15 °C (105.2 MPa). The values of FS in the present study ranged from 46.5 MPa (seT PP for 8 °C) to 120 MPa (Bifix Hybrid Abutment), which is consistent with the results obtained in the study by Kumbuloglu et al., where flexural and compressive strength and surface microhardness of four composite cements (Panavia F, Variolink 2, Reny Unicem Applicap and RelyX ARC) and one polycarboxylic cement (Durelon, control group) were evaluated after storage in water for 1 week. Variolink 2 had the highest flexural strength (90 MPa), while Durelon had the lowest (28 MPa) [28].

Previous studies on composite materials used in restorative dentistry indicate that the three-point flexural strength is closely related to the content of the filler and increases with a higher amount of this filler; however, heating the material itself has little effect on an increase in this value in these materials [24,29,30]. The filler content of Bifix Hybrid Abutment cement is 71% by weight, and the obtained value of 94–120 MPa in three-point flexural strength over the entire temperature range tested confirms this study, given that the other cements tested had a lower filler content, i.e., Multilink Automix—40%, seT PP—60% and MaxCem—60%. In the present study, it was found that changes in conditioning temperature did not significantly change the FS in any of the cements tested. This indicates that storage temperature between 8–35 °C has no effect on strength properties of the tested dual cements. There were no statistical differences found in the FM values for two of the studied cements (seT PP, MaxCem). In the case of the other two tested materials, some significant differences were observed. This may result from imperfections in the research method used, namely the lack of extensometers that allow for accurate measurement of the deformation during the procedure. However, considering the FS and DTS results, it may be assumed that these changes in FM did not affect strength properties.

**Figure 1:**
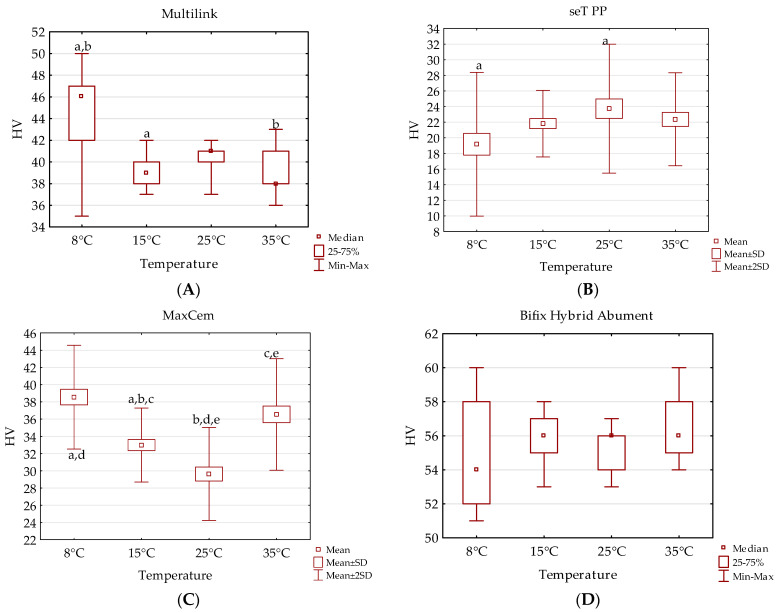
Dependence of hardness HV as a function of storage temperature for different cements: (**A**) Multilink, (**B**) seT PP, (**C**) MaxCem, (**D**) Bifix. The same letters indicate a statistical difference (*p* < 0.05).

Hardness tested at four different conditioning temperatures reached the highest values for the Bifix Hybrid Abutment cement, whereas at the temperature of 8 °C, the value was 54, and at the temperatures of 15 °C, 25 °C and 35 °C, the HV was 56. However, the lowest hardness (HV) values at all of the tested temperatures were obtained with the seT PP cement (8 °C—19, 15 °C—22, 25 °C—24, 35 °C—22). The most significant statistical differences in hardness between the tested groups were found for MaxCem and Multilink Automix. Unfortunately, the observed changes may have resulted from the less homogeneous morphology of dual cements (less reproducible results for material properties) due to mixing in a syringe. Another difference in the above-mentioned materials was the lack of silica in their composition. Silica, as a filler with a smaller particle size, may be more easily and better distributed in bulk as compared to other, different fillers, e.g., glass. Hardness, as reported in the available studies, is strictly dependent on the volume content of the filler, as well as the size and shape of the filler particles. It determines the resistance of a given material to the indentation used on its surface [31]. The value of hardness may be considered as an indication for evaluating the mechanical strength of a given material. The hardness in the conducted test reached the highest value for Bifix Hybrid Abutment cement, which can be explained by the highest volume content of filler in this cement (70% by weight) (Figure 1).

It is noteworthy that the four dual composite cements used in our study differed in the composition of their polymer matrix. Bifix Hybrid Abutment cement used urethane dimethacrylate (UDMA), MaxCem used 2,2-bis [4-(2-hydroxy-3-methacryloxypropoxy)-phenyl]propane) (Bis-GMA), and Multilink, apart from the basic monomer, also used a comonomer in the form of 2-hydroxyethyl methacrylate (HEMA). Multilink Automix exhibited poor chemical adhesion and contained no adhesive monomers. It is considered a durable resin cement [32]. SeT PP contained urethane dimethacrylate (UDMA) in its composition. Modern composite materials used for filling cavities, as well as composite cements, are composed of monomers that are divided into two types, i.e., basic and comonomers. Basic monomers have a high molecular weight, and their function is to crosslink the polymer matrix to give the filling the appropriate shape and mechanical properties. Basic monomers include only the following: bisphenol A ethoxylated dimethacrylate (Bis-EMA), Bis-GMA and UDMA, In contrast, comonomers used in composite materials include 2-hydroxyethylene methacrylate (HEMA), ethylene monoglycol dimethacrylate (EGDMA), ethylene diglycol dimethacrylate (DEGDMA) and triethylene glycol dimethacrylate (TEGDMA). The comonomer’s function is to attenuate and disperse the base monomer, and its presence affects the polarity of the material. Composite materials based on the basic monomer alone have good mechanical properties; however, the addition of comonomer probably indirectly results in better distribution of the filler, owing to the dispersion function of the basic monomers [33,34,35,36,37]. In the present study, among all four cements tested, only Multilink contained comonomer (HEMA). It also had the lowest filler content (40%) and showed hardness values (HV) in the range of 38–46, which places it in the second position in terms of HV, right after the material with the largest share of the inorganic phase. Adding comonomer may have a decisive effect on the uniform distribution of a filler, owing to the dispersion function of the basic monomer. The study by Szczesio-Wlodarczyk et al. found that addition of HEMA monomer has a positive effect on FS and FM [38]. This beneficial phenomenon can be explained by the better mobility of bis-GMA and UDMA in HEMA, and may result from the fact that HEMA has a lower viscosity and better mobility than TEGDMA. Therefore, it could also translate into a higher conversion degree. This observation may explain the fact that composites containing HEMA exhibit better strength properties [38]. Also, it may further be assumed that the hardness of the composite cement could be influenced by both the volume content of the filler and the polymer matrix and the presence of the comonomer as well. This would imply that the hardness of the composite cement is not only affected by the volume content of the filler but also by the addition of comonomer, which uniformly disperses the filler particles, thus improving the hardness. However, studies including a larger number of samples would be required to confirm this theory. If these suppositions are confirmed, the mechanical properties of composite cements could be further improved.

Usually, dual-cured resin cement is chemically set by a reaction of benzoyl peroxide with aromatic tertiary amines. These compounds are susceptible to degradation during storage, which may influence the setting mechanism of cements and their final properties [39]. The study also tested the Set PP material, which is referred to as a dual composite. However, considering the components of this material, it can be assumed that it should be classified as a resin-modified glass ionomer cement. In general, chemical reactions are known to be accelerated by heat. It was shown that significant changes occurred in the working time/setting time of traditional and resin-based cements at higher temperatures [40]. T.A. Pegoraro et al. found that a material set at a lower temperature exhibited a longer setting time than those set at elevated temperatures [41]. However, in this study, the materials were polymerized using light, which made their chemical setting very limited (both in the case of RMGIC and dual-cured cements) [42,43]. Additionally, during the curing of resin materials, heat is generated [43]. This may explain the observed results, i.e., no significant differences could be seen despite the starting temperature of the materials. It is possible that the thermal effects that occur very quickly during photopolymerization must equalize the temperature of the material. However, taking into account the stability of the components of the materials during storage in the laboratory, as well as the extension of the initial working time with cement, keeping dual cements at lower temperatures may bring some positive effects [41].

## 5. Conclusions

Considering the obtained results and the limitations of the study, the following conclusions can be drawn:

1. Strength properties (DTS, FS) were not dependent on storage temperature in the range of 8–35 °C.
2. There were some fluctuations in the hardness of MaxCem and Multilink Automix during storage at different temperatures; however, more extensive analysis is needed to determine if these observations were not coincidental.
3. The cement with the highest filler content (70% by weight) showed the highest values for three-point flexural strength (FS) and the highest values for hardness (HV).
4. Further studies should be carried out to verify whether materials are influenced by storage temperature when light curing is limited.

## Figures and Tables

**Table 1:** The composition of the investigated resin cements.

Cement	Polymer Matrix	Fillers	Filler Content
Multilink Automix (Ivoclare Vivadent)	Dimethacylate, 2-hydroxyethylomethacrylate (HEMA)	Inorganic fillers, barium glass, ytterbium trifluoride, sferoid mixed oxide.	The total inorganic filler is approximately 40% by volume/69% by weight.
seT PP (SDI)	Urethane dimethacrylate > 20% (UDMA), camphorouinone > 1%, acid monomer > 20%,	Fluoroaluminosilicate glass (60%).	The total inorganic filler is approximately 65% by weight.
MaxCem (Kerr)	1,6—heksanediyl bismethcrylete, 2-hydroxy—1,3—propanediyl bismethacrylate, 7,7,9 (or 7,9,9)—trimethyl—4,13—dioxo—3,14—dioxa—5,12—diazeheksadecane—1,16—diylbismethacrylate, 3—trimethoxysilylpropyl methacrylates (Bis-GMA)	Barium aluminoborosilicate glass, ytterbium fluoride, fumed silica.	The total inorganic filler is approximately 46% by volume/65% by weight.
Bifix Hybrid Abutment (Voco)	Urethane dimetacrylate (UDMA), glycerin dimethacrylaate, catalyst, Initiator, alkohol silan methacrylates, phosphoric acid methacrylates and sulphide methacrylates	Fumed silica.	The total inorganic filler is approximately 71% by weight.

**Table 2:** Measurement results for three-point flexural strength (FS), flexural modulus (FM), diametral tensile strength (DTS), and Vickers hardness (HV) of Multilink Automix cement. The same lowercase letters indicate a statistical difference at the level of *p* ≤ 0.05.

	Temperature [°C]	FS [MPa]	SD	FM [MPa]	SD	DTS [MPa]	IQR	HV [-]	IQR
Multilink Automix	8	98.1	15.8	5603 ^a,b^	535	44.1	3.9	46 ^a,b^	5
	15	105.2	13.9	6720 ^a,c^	313	45.2	2.8	39 ^a^	2
	25	91.8	14.8	5273 ^c,d^	520	46.2	2.9	41	1
	35	93.8	17.5	6534 ^b,d^	296	46.2	3.6	38 ^b^	3

**Table 3:** Measurement results for three-point flexural strength (FS), flexural modulus (FM), diametral tensile strength (DTS), and Vickers hardness (HV) of seT PP cement. The same lowercase letters indicate a statistical difference.

	Temperature [°C]	FS [MPa]	SD	FM [MPa]	IQR	DTS [MPa]	IQR	HV [-]	SD
seT PP	8	46.5	14.7	2300	860	44.3	4.0	19 ^a^	5
	15	60.3	12.0	2850	1070	44.8	4.3	22	2
	25	56.2	21.0	2570	1060	45.5	10.3	24 ^a^	4
	35	63.4	14.0	3070	1350	40.9	7.6	22	3

**Table 4:** Measurement results for three-point flexural strength (FS), flexural modulus (FM), diametral tensile strength (DTS), and Vickers hardness (HV) of MaxCem cement. The same lowercase letters indicate a statistical difference.

	Temperature [°C]	FS [MPa]	IQR	FM [MPa]	IQR	DTS [MPa]	SD	HV [-]	SD
MaxCem	8	73.8	23.2	5110	1580	52.8	4.5	39 ^a,d^	3
	15	80.4	10.8	5950	570	56.3 ^a,b^	4.3	33 ^a,b,c^	2
	25	73.1	14.6	5032	390	49.7 ^a^	5.2	30 ^b,d,e^	3
	35	85.8	33.1	6110	550	49.0 ^b^	7.4	37 ^c,e^	3

**Table 5:** Measurement results for three-point flexural strength (FS), flexural modulus (FM), diametral tensile strength (DTS), and Vickers hardness (HV) of Bifix Hybrid Abutment cement. The same lowercase letters indicate a statistical difference.

	Temperature [°C]	FS [MPa]	SD	FM [MPa]	IQR	DTS [MPa]	IQR	HV [-]	IQR
Bifix Hybrid Abutment	8	109	11	8450	1010	43.9	20.51	54	6
	15	113 ^a^	9	8760 ^a^	765	48.4	10.49	56	2
	25	94 ^a,b^	13	6050 ^a,b^	1845	54.8	9.63	56	2
	35	120 ^b^	12	8640 ^b^	260	46.7	9.85	56	3

## Data Availability

Data are available in a publicly accessible repository Zenodo at: https://zenodo.org/record/8365737 (accessed on 18 September 2023).
